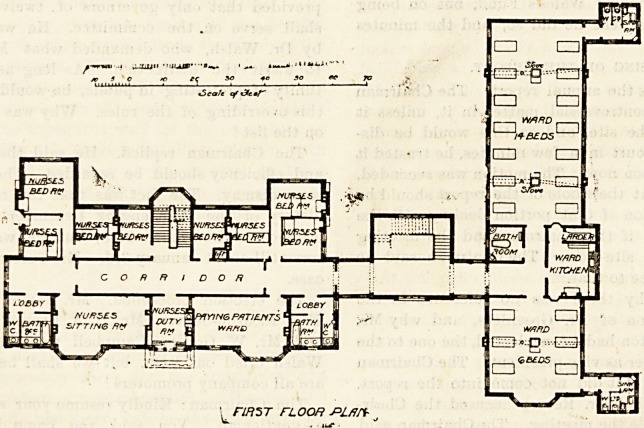# Victoria Hospital, Folkestone

**Published:** 1902-04-19

**Authors:** 


### The Institutional Workshop.

### VICTORIA HOSPITAL, FOLKESTONE.

The older part of this hospital was built in commemoration of the 1887 Jubilee, and it is now used chiefly for administrative purposes. The memorial stone of the new wing, of which we give the ground and first floor plans, was laid by the Duke of Cambridge in June, 1900. The six-bedded ward and the main staircase are not built yet, but the work will be undertaken as soon as the funds are forthcoming. The large wards and the administrative part are finished. The building is bisected by a wide corridor, at one end of which is placed a ward containing 14 beds. The ward is well designed; each bed has a window on both sides; there is a large window in the end of the ward, and the sanitary block is properly constructed, and is effectually cut off from the ward by a cross-ventilated passage. Between the ward end of the corridor and the ward itself is the bath-room, and opposite the bath-room is a good ward-kitchen and larder.

The floors are of teak, and the wards are warmed by Shorland's grates. The walls are plastered, and painted with enamel. The baths are of white vitreous enamelled iron. A considerable length of corridor separates the wards from the administrative department. This is very desirable. On one side of the corridor are the committee-room, secretary's office, visitors' room, staircase and porter's room, and nearest to the ward are the operating theatre and anæsthetic room. On the other side of the corridor are bath-room, house-surgeon's rooms, vestibule, matron's room, paying patients' ward and recovery room.

The large wards on the first floor are the same as those of the ground floor; and over the board-room and the other rooms on the same side of the corridor, are the nurses' bedrooms; while on the opposite side of the corridor are the bath-room, closet, nurses' sitting-room, and nurses' duty-room. The latter is next to the paying patients' ward, and at the other end of the latter is a lobby, approached from the corridor, and leading to bath-room and closet. The paying patients' ward has a good bay window, and another window, but three of its sides are blocked.

The architect was Mr. H. Percy Adams. The contractors were Messrs. Gough and Co., of Hendon, and the building cost £6,000. It redounds greatly to Mr. Adams' credit that there were no extras.

## Figures and Tables

**Figure f1:**
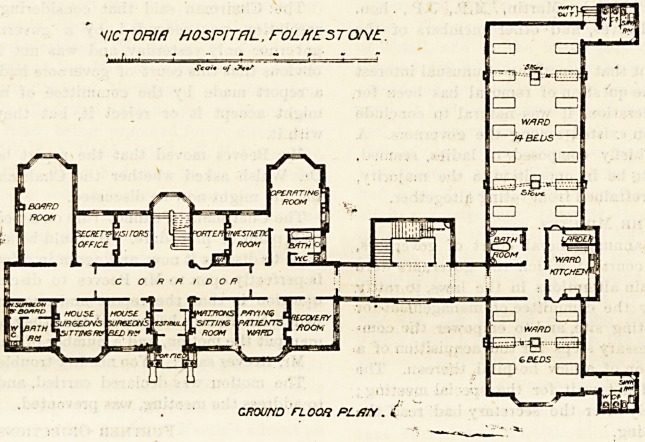


**Figure f2:**